# Inequal distribution of nursing personnel: a subnational analysis of the distribution of nurses across 58 countries

**DOI:** 10.1186/s12960-022-00720-5

**Published:** 2022-03-05

**Authors:** Mathieu Boniol, Carey McCarthy, Deen Lawani, Gilles Guillot, Michelle McIsaac, Khassoum Diallo

**Affiliations:** Health Workforce Department, World Health Organization, Geneva, Switzerland

**Keywords:** Nursing, Health workforce, Healthcare disparities, Demography

## Abstract

**Background:**

Nursing personnel are critical for enabling access to health service in primary health care. However, the State of the World’s Nursing 2020 report showed important inequalities in nurse availability between countries.

**Methods:**

The purpose of this study/analysis was to describe the differences in nurse-to-population density in 58 countries from six regional areas and the relationship between differences in access to nurses and other indicators of health equity.

**Results:**

All countries and income groups showed subnational inequalities in the distribution of nursing personnel with Gini coefficients ranging from 1 to 39. The latter indicated situation such as 13% of the population having access to 45% of nurses in a country. The average max-to-min ratio was on average of 11-fold. In our sample, the African region had the highest level of subnational inequalities with the average Gini coefficient of 19.6. The European Region had the lowest level of within-country inequalities with the average Gini coefficient being 5.6. A multivariate analysis showed a clustering of countries in three groups: (1) high Gini coefficients comprised mainly African countries; (2) moderate Gini coefficients comprised mainly South-East Asian, Central and South American countries; (3) low Gini coefficients comprised mainly Western countries, Japan, and Korea. The analysis also showed that inequality in distribution of nurses was correlated with other indices of health and inequality such as the Human Development Index, maternal mortality, and life expectancy.

**Conclusions:**

This study showed that there is a high level of geographic inequality in the distribution of nurses at subnational level. Inequalities in nursing distribution are multifactorial, to improve access to nurses, policies should be bundled, tailored to the local context and tackle the various root causes for inequalities.

**Supplementary Information:**

The online version contains supplementary material available at 10.1186/s12960-022-00720-5.

## Background

Achieving Universal Health Coverage (UHC) through primary health care (PHC) is the principal mechanism for achieving Sustainable Development Goal (SDG) 3: *Ensure healthy lives and promote well-being for all* [1–3]. The World Health Organization (WHO) monitors progress towards UHC by the level and equity of coverage in countries as the proportion of a population that can access essential quality health services [4]. Even while 2019 assessment found that overall coverage was increasing, they found that inequality persisted, poor countries were lagging behind and within-country coverage varied substantially; leaving some of the most vulnerable behind [5]. Since the relationship between health workers and health outcomes is well established, and as part of SDG objective 3.c, WHO also tracks progress in access to health care workers [6]. Development of the WHO Global Strategy for Human Resources for Health (GSHRH) identified important global disparities in health workforce availabilities and a shortage of up to 18 million health workers, primarily in low and middle-income countries [7].

In many countries, the population need for health workers is not matched by economic demand and ability to pay for health worker jobs, nor by the technical and financial resources to produce the necessary health workforce [8, 9]. Undoubtedly, social, economic, and political policies can also influence the extent countries to invest in human resources for health [10, 11]. The biggest gaps are in least developed countries and small island developing states where PHC spending per capita and investments in human resources for health need to substantially increase [9]. A challenge for countries from all income groups is ensuring the availability of valid, reliable, up-to-date, and easily accessible data on human resources for health with which to make national health policy decisions [12, 13]. While the number of countries able to report standardized national health workforce data is increasing, countries must have these data at a subnational level to ensure equitable distribution for UHC, leaving no one behind [14–17].

Nurses can greatly contribute to equitable service coverage and access to health workers through efficacious and cost-effective primary health care and non-communicable disease services [18–25]. Nurses in advanced practice roles can improve the quality and efficiency of care as well as greatly increase access to primary health care services [26–30]. However, the *State of the World’s Nursing 2020* (SoWN) report identified vast inequities in the density and distribution of nurses globally, across WHO Regions, and between countries within Regions [31]. During the development of the State of the World’s Nursing report, relatively few countries reported their subnational nursing headcounts, suggesting it was not available for health workforce and health services planning. While some studies of health workforce density and distributional equality have included nurses [32–34], there have not yet been dedicated studies examining how the subnational density of nurses relates to equality in service coverage and other indicators of health equity relevant to UHC.

The purpose of this study was to identify the differences in nurse-to-population density in a sample of 58 countries from the six WHO Regions, describe the relationship between inequality in the subnational distribution of nurses and other indicators of inequality, and discuss relevant policy approaches to advance towards UHC.

## Methods

Data on the number of nursing personnel at the first administrative level were obtained from two sources. First, data reported to WHO as part of the elaboration of the State of the World Nursing report 2020 [31] were gathered through the National Health Workforce Accounts (NHWA) [35]. These data were complemented by census data obtained from the Integrated Public Use Microdata Series (IPUMS) database [36] for countries reporting sufficient details on occupation of census participants, i.e. a four-digit code established in the 1988 or 2008 revisions of the International Standard Classification of Occupations. Each country was classified according to their income group as per World Bank grouping as of June 2020. Data reported to WHO spanned from 2013 to 2018, and data from latest available census were extracted over the period 2005 to 2017.

Population data were identified for each country and each administrative unit using data reported mostly by national statistical offices (NSO). When there was a difference between the population size from these sources and the population size from the Population Division of the Department of Economic and Social Affairs (UN DESA) of the United Nations Secretariat [37], the population by subnational unit was rescaled to the population of UN Population Prospects. This ensured that subnational densities were reported with a similar denominator as the UN Population Prospects enabling cross-country and within-country comparisons. When year of reporting of count of nursing personnel and population distribution differed, a scaling factor was first applied based on population size in UN population prospect between year of population data and year of nursing data. All sources on the count of nurses and population are provided in supplementary material (Additional file 1: Table S1).

For each country, additional contextual indicators were extracted, including, the Human Development Index (HDI), the share of urban population from UN data division, out-of-pocket health expenditure and current health expenditure (CHE) as percentage of gross domestic product (GDP) from the WHO’s Global Health Observatory of World Health Organization [38].

For each country and each subnational level, the density of nursing personnel was expressed as the number of nursing personnel, defined as headcount, per 10,000 population. The number of subnational units were reported. The inequality in the distribution of nursing personnel was measured by two indicators. The ratio of the administrative unit with highest density of nurses to 10,000 population to the administrative unit with the lowest density (max-to-min ratio) in a country was calculated. There are many metrics that can be calculated to reflect distributional inequality of health workforce across geographic units [39]. The metrics employed in this study are the max-to-min ratio and the Gini Index. The density of nurses to populations at subnational units was considered as a resource in the computation of a Gini coefficient. Hereafter, mentions of “Gini coefficient” correspond to this measure of geographic inequality in nursing workforce distribution across the first administrative unit in a given country. This Gini coefficient here is expressed as a percentage from 0 to 100; this can be interpreted as the magnitude of deviation from an hypothetical equal distribution of nurse-to-population ratio across all subnational units. Values close to 0 indicate a situation of equality. The Gini coefficient has the advantage of measuring the degree of distributional inequality in nurse-to-population density across all administrative units, whereas the max-to-min ratio only used data from two regions.

Because the number of subnational units varies across countries (range: 3–54), it is plausible that countries with more administrative units would have larger max-to-min ratio due to more granular distribution of population. Similarly, the Gini coefficient, although using the data from every administrative unit available, could also be biased to smaller value when very few administrative units were available; therefore inaccurately reflecting the degree of geographical inequality. To assess the degree of bias by the number of units in the present sample of countries, the two measures of inequality were compared with the number of administrative units (Additional file 1: Figs. S1 and S2). This showed that comparison across countries in this sample for this study did not seem systematically biased by the number of administrative units in countries. Nevertheless, caution should be employed when comparing such measures across countries.

Boxplots were used to describe the distribution of the Gini coefficient by the World Bank income classification.

### Statistical modelling

The dataset of contextual socio-economic indicators contained missing entries, therefore, to enable inclusion in the analysis of all countries, missing entries were imputed. This was done by training a Random Forest algorithm then by predicting missing entries [40]. Countries were clustered into groups, using all additional contextual indicators described above except the Gini coefficient for subnational distribution of nurses. The number of clusters considered in the analysis varied between two to six. The clustering technique was based on Gaussian mixture models with the assumption that the rows of the data matrix represented points centered around cluster-specific ellipses in a multi-dimensional space. Lastly, to investigate more closely the relation between the Gini coefficient and other socio-economic variables, a multivariate analysis was conducted using a (beta) regression of the Gini coefficient on other factors listed above as independent variables.

## Results

Data on nursing personnel distribution were obtained from 58 countries representing a wide range of countries from all income groups and all WHO Regions, and from countries with very low density of nursing personnel, such as Madagascar with only 1.5 nursing personnel per 10,000 population, to countries with high density, such as Ireland with 164.7 nurses per 10,000 populations (Table 1).

**Table 1:** Summary of nursing density and subnational distribution statistics for 58 countries

Country	National density of nursing personnel*per 10,000 population	(Min–max)	Number of subnational units	Amplitude of variation (max/min)	Gini index (0–100)
Afghanistan	2.5	(0.8–11.1)	34	14.4	20
Angola	4.1	(2.5–8.1)	18	3.3	21
Armenia	48.4	(21.8–62.2)	11	2.9	11
Australia	116.9	(103–150.3)	8	1.5	4
Belarus	99.9	(95.8–106.7)	6	1.1	2
Belize	23.4	(14.5–33.4)	6	2.3	13
Bolivia	15.6	(8.6–28.6)	9	3.3	3
Brazil	38.5	(21.2 – 55.0)	25	2.6	8
British Virgin Islands	92.6	(31.1–104.4)	4	3.4	11
Burkina Faso	5.4	(2.8–11.9)	13	4.2	19
Cambodia	6.9	(2.7–22.2)	25	8.3	4
Congo	7.5	(3.4–15.3)	12	4.5	9
Cook Island	66.8	(48.2–179.2)	12	3.7	2
Cote d'Ivoire	6.0	(4.1–15.6)	19	3.9	6
Cuba	75.6	(68.7–98.6)	14	1.4	1
DRC	5.4	(1.3–77.2)	26	60.3	26
Ecuador	28.3	(17.9–40.3)	24	2.3	3
Egypt	58.3	(24.4–179.8)	24	7.4	9
El Salvador	13.7	(3.1–20.2)	14	6.4	21
Eritrea	12.1	(4.9–34.6)	6	7.1	32
Eswatini	37.1	(24.2–53.5)	4	2.2	15
Ethiopia	5.4	(2.5–22.5)	11	9.0	15
Fiji	6.8	(2.2–11.6)	4	5.2	30
France	84.4	(57.7–109.1)	26	1.9	2
Greece	38.1	(12.5–70.7)	54	5.6	13
Guyana	9.4	(0.4–17.1)	10	44.1	7
Haiti	3.8	(1.5–7.7)	4	5.2	1
Honduras	7.3	(2.5–31.9)	18	12.5	9
India	8.6	(1.8–329.6)	29	179.0	8
Iran	20.0	(12.7–28.2)	30	2.2	2
Iraq	20.4	(11.2–39.1)	18	3.5	4
Ireland	164.7	(134.2–202.8)	8	1.5	1
Japan	121.5	(83.6–199.5)	47	2.4	8
Kenya	11.7	(2.1–33.3)	47	15.5	29
Korea (Republic of)	73.0	(2.6–105.3)	17	40.7	6
Lesotho	17.8	(7.1–33.3)	10	4.7	22
Madagascar	1.5	(0.4–9.8)	22	26.2	39
Malawi	4.4	(4.1–4.7)	3	1.1	1
Malaysia	34.7	(21.8–342.0)	16	15.7	3
Mali	4.5	(1.8–16.2)	8	9.2	39
Mexico	24.2	(11.8–41.7)	32	3.5	13
Micronesia	20.4	(12.6–44.9)	4	3.6	7
Mozambique	4.8	(3.5–8.2)	11	2.3	6
Nigeria	13.6	(2.5–34.9)	24	13.9	34
Pakistan	7.5	(1.3–21.3)	6	16.4	1
Panama	29.5	(8.1–41.1)	7	5.1	5
Peru	23.7	(16.4–50.0)	24	3.1	6
Philippines	49.4	(28.7–72.5)	13	2.5	2
Portugal	58.8	(32.3 – 152.0)	22	4.7	3
Romania	56.2	(17.7–89.3)	39	5.0	7
South Africa	13.1	(7.8–16.6)	9	2.1	6
United Arab Emirates	57.3	(26.0–84.2)	7	3.2	21
United States of America	109.6	(30.0–152.1)	51	5.1	3
Uruguay	29.9	(13.2–39.5)	19	3.0	15
Vanuatu	14.2	(9.8–22.8)	4	2.3	10
Venezuela	20.7	(1.9–44.8)	24	23.3	3
Vietnam	16.8	(8.4–24.1)	38	2.9	11
Zambia	9.9	(5.0–13.9)	10	2.8	16

*Defined as headcount

A broad diversity in distribution of nursing personnel at subnational level could be seen with the max-to-min ratio. It ranged from 1.1 in Belarus to 179 in India. The stunning variation across countries of the max-to-min ratio statistics could be due to extreme densities in very small populations, and therefore might be of limited significance from a global perspective. These should be interpreted with caution as they have a rather low correlation (*R*^2^ = 11% Pearson correlation) with Gini index describing inequality over the whole population (Additional file 1: Fig. S3).

The Gini index ranged from 1—indicating nearly equal distribution of nursing personnel—in Cuba, Haiti, Ireland, Malawi, and Pakistan to 39 in Madagascar and Mali. In Mali, for example, 2950 out of the 6490 nursing personnel in the country were in Bamako, which means that 13% of the population had access to nearly half (45%) of nurses in the country.

The data from these 58 countries indicated that the level of inequality varied by WHO Regions, with lower geographical inequality observed in the European Region and the Region of the Americas (Table 2). The largest inequities were observed in the African Region.

**Table 2:** Average measure of inequality in distribution of nursing personnel by WHO Region

WHO Region	Number of countries (total)	Average number of subnational units	Average max/min ratio	Average Gini index
African Region	17 (47)	15	10.1	19.6
Region of the Americas	16 (35)	18	7.9	7.6
Eastern Mediterranean Region	6 (21)	20	7.8	9.5
European Region	7 (53)	24	3.3	5.6
South-East Asia Region	1 (11)	29	179.0	8.2
Western Pacific Region	11 (27)	17	8.1	7.8
Total	58 (194)	18	11.0	11.1

**Fig. 1:**
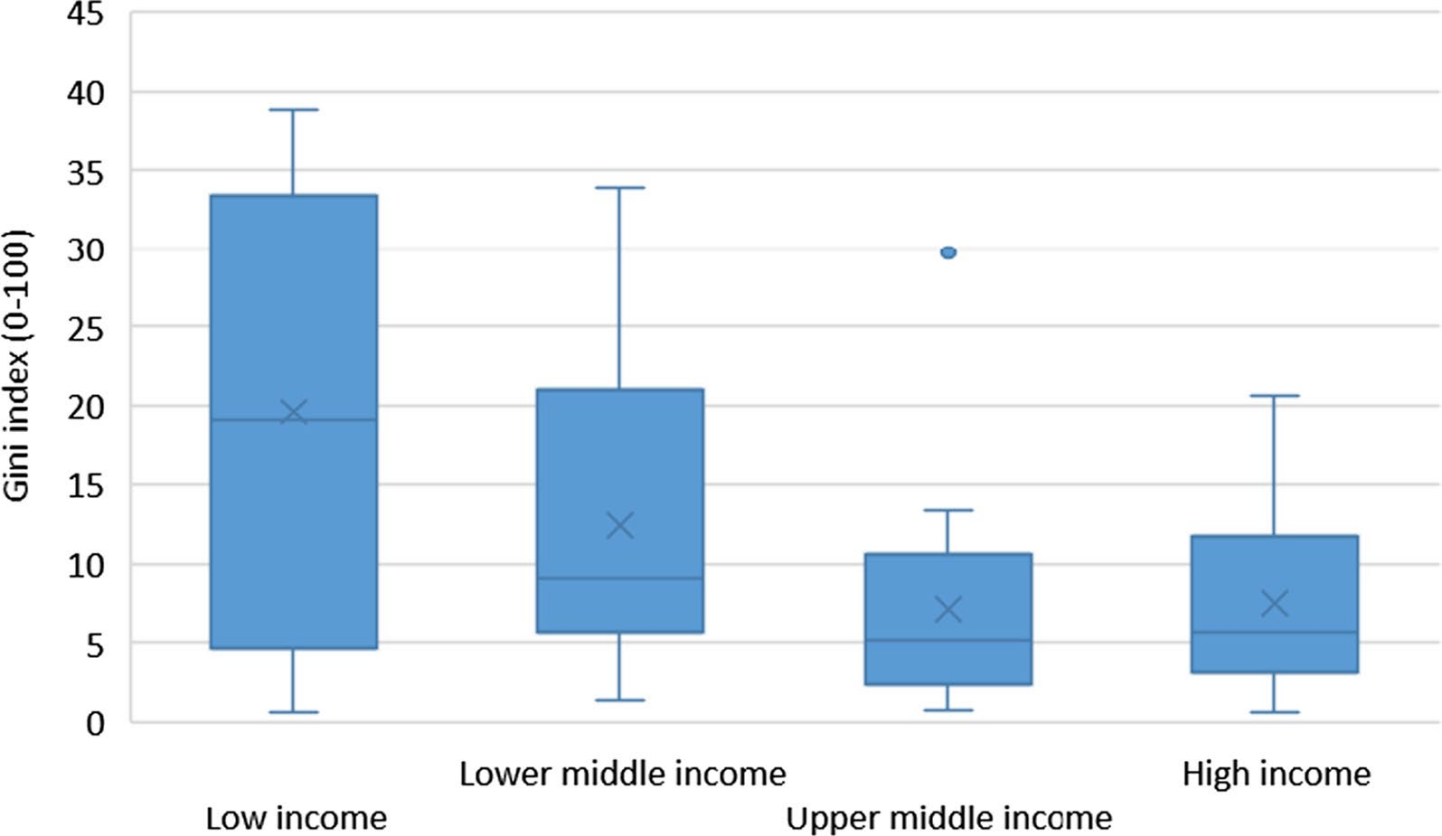
Boxplot of the distribution of Gini index of the subnational distribution of nursing personnel (Gini coefficient close to 0 means equal distribution of nurses across regions) in 58 countries by income group

Countries in the highest income category, either from upper-middle income or high-income, had a lower average Gini coefficient (on average 7.4) than countries from lower-middle (on average 12.4) or low-income group (on average 19.6) (Fig. 1).

**Fig. 2:**
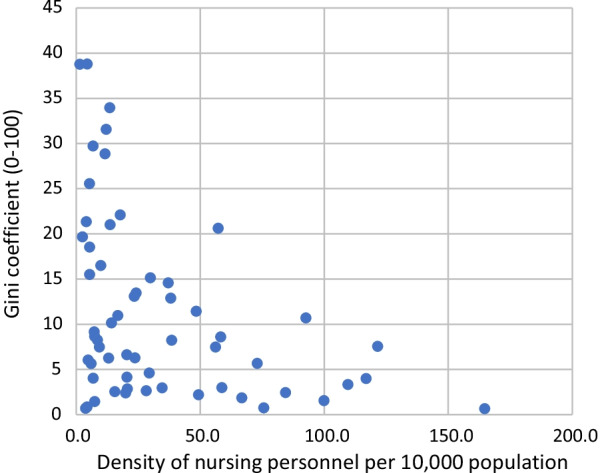
Scatter plot of the Gini coefficient (0–100) and density of nursing personnel per 10,000 in 58 countries

High Gini coefficients were mostly seen in countries with low density of nursing personnel (Fig. 2), while for most countries with a density of nursing personnel above 25 nurses per 10,000 population the Gini coefficient only rarely exceeded 15% (only in United Arab Emirates and Venezuela).

Similar to the World Bank income classification, a correlation was observed between the average Gini coefficient and the Human Development Index (*R*^2^ = 24%): the higher the development index the lower was the Gini coefficient. Other factors such as the health expenditure were only weakly correlated with Gini coefficient of nursing density such as health expenditure (*R*^2^ = 9.7%). Lastly, the level of national urbanization, as the share of total population living in urban area, was not correlated with the inequitable distribution of nursing personnel (*R*^2^ = 1.8%).

**Fig. 3:**
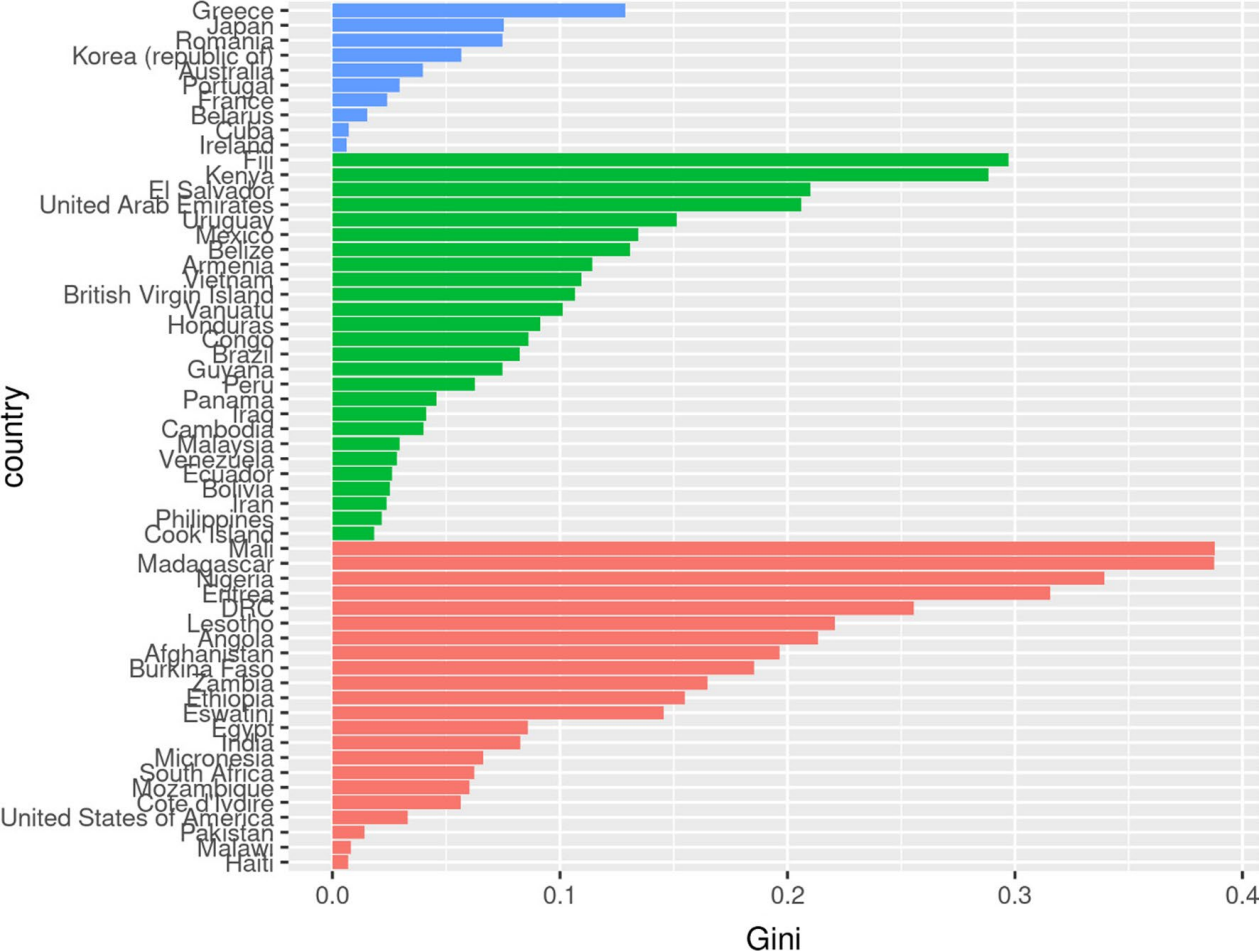
Variation of Gini coefficient (express here as 0–1) of nurse density at subnational level. Country clusters were obtained based on socio-economic indicators only. *Note* The indicators included in the clustering analysis are available in supplementary material

The analysis of data through clustering models revealed a clustering of countries into three groups: a first group (in red on the figure) with countries mainly from the African region, a second group (in green) consisting of countries mainly from the South-East Asia region, Central and South American countries, and a third group (in blue) consisting predominantly of upper-middle and high-income countries, Japan, and Korea (from bottom to top in Fig. 3).

Variation of the Gini coefficient was group-specific, with values of the Gini coefficient shifted towards lower values from group 1 (African countries) to group 3 (European countries). It was also noticeable that the ranges overlapped, for example with Gini coefficient of Greece (0.13, group 3) of the order of that of Mexico (0.13, group 2) or Ethiopia (0.15, group 1). The United States of America was clustered in the first group which consists mostly of African countries, but had a Gini coefficient of 0.03 which is close to the mean value for the group of European countries. In a sensitivity analysis allowing aggregation of countries into four groups, the United States of America becomes an outlier and included as a separate group. The multivariate analysis with beta regression showed the strongest association of the Gini coefficient with four variables: the human development index, the maternal mortality ratio per 100,000 live births, the inequality in life expectancy and the current health expenditure as percentage of GDP (Additional file 1: Table S2).

## Discussion

The analysis of subnational distribution of nursing personnel in 58 countries revealed inequity in the distribution of nurses within countries. Disparities were observed across all WHO Regions and all income groups although the largest inequalities in the distribution of nursing personnel were found in the African region. Countries with lowest densities of nursing personnel tend to have largest Gini coefficient. On average, an 11-fold difference was observed between the regions with highest nurse densities as compared to the region with lowest densities. Therefore, a similar magnitude of distributional inequality exists at subnational level as compared to the global and regional differences identified in the SoWN 2020. The largest inequalities found in the present analysis were in countries in the African Region which simultaneously have the lowest overall densities of nursing. These countries face both a scarcity of nursing personnel at national level and a severe maldistribution of the few nursing resources within country. While the size of the global nursing workforce is projected to grow by approximately 8 million by 2030, approximately 70% of the growth is expected in upper middle- and high-income countries [31]. The SoWN 2020 estimated that low- and lower-middle income countries would need to increase their domestic production of nurses by about 8% per annum, alongside sustained investments in the recruitment and retention of the graduates, at a cost of approximately USD $10 per capita per year through 2030 [31].

Effectively and sustainably increasing investments in health worker education and employment requires multisectoral policy action. The GSHRH [7] uses a health labour market framework [41] to describe policy options that can improve the recruitment, development, training and retention of the health workforce, with specific considerations for least developed countries and small island developing states. The retention of health workers in rural or remote area is a pervasive challenge, but several, acceptable and feasible, policy inventions have been identified [42, 43]. Policy measures tailored to the local context that simultaneously address education and training, regulation, incentives and support in an integrated and bundled way are proven to help reduce the geographic maldistribution of health workers [44]. Investments and policies must include safeguarding and protection of health workforce [45, 46].

The Global Strategic directions for nursing and midwifery 2021–2025 [47] provides policy priorities, aligned with the guidance above, to ensure that nurses maximally contribute to national health goals and UHC. Policy-relevant evidence on what specifically contributes to nurses’ retention in health and care settings [48–51], including for advanced practice nurses in rural and remote settings, is increasingly available [52–54]. Institutionalizing these aspects requires a context-specific aligning of the education, training, and lifelong learning of nurses with optimized roles of nurse in the health system, updating and strengthening professional regulations, providing leadership and career advancement opportunities, ensuring safe and supportive service delivery environments, and competent management, planning and forecasting of the nursing workforce to ensure nurses are where they are most needed [31, 47].

The multivariate analysis identified a variety of factors potentially associated with this inequitable distribution of nursing personnel, with inequity more important in countries with lower human development index, with high maternal mortality, and high inequity of life expectancy. It should be noted that this comparison is based on data aggregated at national level, and further investigation should require all these factors being available at subnational level. There was clear opposition between higher income countries (Western Europe, Japan, Korea) with high development index and higher expenditure in education, and the grouping of countries comprising lower income countries of Asia, Central and South America. This could suggest that weaker investment in the human capital both for the active workers and for the education and that existing inequities in these areas results in inequitable distribution of nurses.

This observation is in line with the 2021 UHC Monitoring Report which found that the within-country relationship between inequalities in accessing essential health services and both the education and urbanization levels was particularly pronounced in low-income countries [55]. It also supports studies which, using subnational data, identified inequalities in the distribution of health workers, correlation with health spending, and impacts on PHC service delivery and health outcomes [56–58]. Related frameworks have also suggested that inequities in various domain are intrinsically correlated and that inequities tend to cluster in particular in the poorest countries as having similar root causes [42].

Identifying root causes of inequities, correlated factors, and impacts upon health and well-being on the route towards UHC requires investment and political commitment to enhance country health information systems for timely, accurate, and disaggregated data [55]. For essential health services to be equitably available, health workforce data must be available at the subnational level. This analysis demonstrated the feasibility to monitor and report the distribution of nursing personnel at the first administrative level for 58 countries. Through the progressive implementation of the National Health Workforce Accounts [35], countries can be better equipped to conduct such evaluations to inform health policy [31, 59, 60]. Senior government nursing leaders should be enabled to and accountable for driving the national agenda to improve and utilize nursing workforce data for health policy decision-making [31, 47].

The present study has a series of limitations, but nevertheless, presents an important step in harmonizing approaches to understanding geographical mal distribution among nurses. While data for countries on all continents could be identified, with only 58 countries included, not enough data on nursing personnel distribution at subnational level exist to provide a comprehensive global description of nursing personnel inequity. These data were also from different points in time, some being rather old relying on census statistics. In addition, they cover different geographies and sizes therefore spatial heterogeneity is an issue. The progress on NHWA implementation would likely result in improved assessment in the coming years, but countries should improve their monitoring and reporting systems to enable such critical assessment. The definition of subnational level varies between countries with sometimes states, department of region being used as first-level subnational unit. In some countries, these will be administrative units aligned with health planning/services in other they will be less aligned to policy intervention. These variations in definition of subnational level will remain in coming years as the United Nation Statistics Division only recently supported the use of the DegUrba methodology to classify the degree of urbanization enabling better comparison of subnational data [61].

## Conclusions

Geographical inequality in access to nursing personnel has been long known between countries [31]. This study shows that geographical inequalities were largely present within countries and that there is an important gradient of inequality; with the poorest countries and those with the most severe shortage of health workers facing the greatest challenges of maldistribution. Countries in the African region, who already had the lowest densities of nurses, also had the largest inequity resulting in exacerbated consequences for populations in remote and rural areas. Investment in nursing education and employment could help tackle the inequity at subnational level. Intersectoral policy action is needed to redress these gaps and ensure the most vulnerable populations have access to nurses and the critical primary health care services they can provide.

## Supplementary Information

**Additional file 1: Table S1.** Summary of subnational data source for the analysis. **Figure S1.** Scatter plot of amplitude of subnational variation of density (max to min ratio) and number of subnational units. Variation of density is the max to min ratio between the region with maximum density and region with lowest density. **Figure S2.** Scatter plot of Gini index measuring the inequitable distribution of nursing personnel and number of subnational units. Variation of density is the max to min ratio between the region with maximum density and region with lowest density. **Figure S3.** Correlation between the Gini index and the Max/min ratio of density of nursing personnel. Gini index and max/min ratio are on a log scale and the dashed line is a power function regression between the two indicators (equation and R^2^ displayed on the graph). **Table S2.** Results of the multivariate beta regression of the Gini index measuring inequality in nursing distribution at subnational level with several socio-economic factors. **Table S3.** List of variables included in the multivariate beta regression, with year, source and data link.

## Data Availability

The datasets used and/or analysed during the current study are available from the corresponding author on reasonable request.

## Abbreviations

CHE: Current health expenditure
GSHRH: Global Strategy for Human Resources for Health
GDP: Gross domestic product
HDI: Human Development Index
NHWA: National Health Workforce Accounts
NSO: National statistical offices
PHC: Primary health care
SDG: Sustainable Development Goal
SoWN: State of the World’s Nursing 2020 report
UHC: Universal Health Coverage
WHO: World Health Organization
