# Supplementary material for: Inequal distribution of nursing personnel: a subnational analysis of the distribution of nurses across 58 countries
Source: Hum Resour Health. 2022 Mar 5;20:22. doi: 10.1186/s12960-022-00720-5 (PMC8898534; doi:10.1186/s12960-022-00720-5)
Supplement: Supplementary file 1 — Additional file 1: Table S1. Summary of subnational data source for the analysis. Figure S1. Scatter plot of amplitude of subnational variation of density (max to min ratio) and number of subnational units. Variation of density is the max to min ratio between the region with maximum density and region with lowest density. Figure S2. Scatter plot of Gini index measuring the inequitable distribution of nursing personnel and number of subnational units. Variation of density is the max to min ratio between the region with maximum density and region with lowest density. Figure S3. Correlation between the Gini index and the Max/min ratio of density of nursing personnel. Gini index and max/min ratio are on a log scale and the dashed line is a power function regression between the two indicators (equation and R2 displayed on the graph). Table S2. Results of the multivariate beta regression of the Gini index measuring inequality in nursing distribution at subnational level with several socio-economic factors. Table S3. List of variables included in the multivariate beta regression, with year, source and data link. [file 12960_2022_720_MOESM1_ESM.docx]

**Supplementary Material**

| **Table S1. Summary of subnational data source for the analysis** | | |  |  |
| --- | --- | --- | --- | --- |
| Country | Nursing data |  | Population data |  |
|  | Source | Year | Source | Year |
| Afghanistan | HRH profile of Afghanistan* | 2018 | Afghanistan NSO (National Statistics and Information Authority, estimated population (2019)) | 2018 |
| Angola | NHWA SoWN | 2018 | Angola NSO (INE) Link: https://www.ine.gov.ao/indicadores-estatisticos/populacao | 2018 |
| Armenia | IPUMS | 2011 | Armenia NSO Link: https://www.armstat.am/file/doc/99486118.pdf | 2011 |
| Australia | NHWA SoWN | 2013-2017 | Australian Bureau of Statistics: Census 2016. Link: https://www.abs.gov.au/websitedbs/D3310114.nsf/Home/2016%20search%20by%20geography | 2016 |
| Belarus | IPUMS | 2009 | Belarus NSO Link: https://www.belstat.gov.by/en/ofitsialnaya-statistika/Demographic-and-social-statistics/population-and-migration/population/annual-data/ | 2009 |
| Belize | NHWA SoWN | 2018 | Belize NSO Link: http://sib.org.bz/wp-content/uploads/2017/05/Census_Report_2010.pdf | 2010 |
| Bolivia | NHWA SoWN | 2015-2017 | Bolivia National Statistics Office. Link:http://censosbolivia.ine.gob.bo/censofichacomunidad/c_listadof/listar_comunidades | 2014 |
| Brazil | IPUMS | 2010 | Brazil NSO | 2010 |
| British Virgin Island | NHWA SoWN | 2015-2018 | Virgin Islands 2010 Population and Housing Census Report | 2010 |
| Burkina Faso | NHWA SoWN | 2015-2017 | Burkina Faso NSO Link : http://www.insd.bf/n/contenu/Tableaux/T0316.htm | 2006 |
| Cambodia | NHWA SoWN | 2013-2018 | Cambodia NSO Link:https://www.nis.gov.kh/nis/Census2019/Provisional%20Population%20Census%202019_English_FINAL.pdf | 2008 |
| Congo | NHWA SoWN | 2015-2016 | Congo NSO Link:http://www.cnsee.org/index.php?option=com_content&view=article&id=135%3Apopdep&catid=43%3Aanalyse-rgph | 2007 |
| Cook Island | NHWA SoWN | 2014-2018 | Cook Island NSO | 2016 |
| Cote d'Ivoire | NHWA SoWN | 2013-2018 | Cote d'Ivoire NSO Link:http://www.ins.ci/n/index.php?option=com_content&view=article&id=19&Itemid=27 | 2014 |
| Cuba | NHWA SoWN | 2013-2018 | Cuba NSO Link: http://www.onei.gob.cu/node/14729 | 2002 |
| DRC | NHWA SoWN | 2017 | DRC NSO | 2014 |
| Ecuador | NHWA SoWN | 2013-2017 | Ecuador NSO Link: https://www.ecuadorencifras.gob.ec/proyecciones-poblacionales/ | 2017 |
| Egypt | IPUMS | 2006 | Egypt NSO | 2006 |
| El Salvador | IPUMS | 2007 | El Salvador NSO | 2007 |
| Eritrea | NHWA SoWN | 2013-2018 | Eritrea NSO | 2000 |
| Eswatini | NHWA SoWN | 2013-2018 | Eswatini NSO Link:https://www.equinetafrica.org/sites/default/files/uploads/documents/SWAZ%20LivingConditions2007.pdf | 2007 |
| Ethiopia | NHWA SoWN | 2017-2018 | Ethiopia Census | 2007 |
| Fiji | IPUMS | 2007 | Fiji Census | 2007 |
| France | IPUMS | 2006, 2011 | France NSO | 2006, 2011 |
| Greece | IPUMS | 2011 | Greece NSO | 2011 |
| Guyana | NHWA SoWN | 2013-2018 | Guyana census Link:https://statisticsguyana.gov.gy/wp-content/uploads/2019/10/2012_Preliminary_Report.pdf | 2012 |
| Haiti | NHWA SoWN | 2018 | Haiti NSO link: http://www.ihsi.ht/pdf/projection/Estimat_PopTotal_18ans_Menag2015.pdf | 2015 |
| Honduras | NHWA SoWN | 2016-2018 | Honduras NSO Link:https://www.ine.gob.hn/V3/ | 2018 |
| India | IPUMS | 2004, 2009 | India NSO | 2009 |
| Iran | IPUMS | 2006, 2011 | Iran NSO | 2006, 2011 |
| Iraq | NHWA SoWN | 2013-2018 | Iraq Link: http://cosit.gov.iq/images/pdf/researches%20ar/6.pdf | 1997 |
| Ireland | IPUMS | 2011 | Ireland NSO | 2011 |
| Japan | NHWA SoWN | 2014, 2016, 2018 | Japan NSO | 2014, 2016, 2018 |
| Kenya | NHWA SoWN | 2013-2018 | Kenya NSO Census report (2019 population and Housing census) | 2019 |
| Korea (republic of) | NHWA SoWN | 2013-2018 | Korea NSO | 2015 |
| Lesotho | NHWA SoWN | 2018 | Lesotho census | 2016 |
| Madagascar | NHWA SoWN | 2018 | Madagascar NSO Link: https://www.instat.mg/accueil/madagascar-en-chiffre/ | 2018 |
| Malawi | NHWA SoWN | 2018 | Malawi NSO Link :http://www.nsomalawi.mw/index.php?option=com_content&view=article&id=226:2018-malawi-population-and-housing-census&catid=8:reports&Itemid=6 | 2018 |
| Malaysia | NHWA SoWN | 2013-2017 | Malaysia NSO Link: https://www.dosm.gov.my/v1/index.php?r=column/ctheme&menu_id=L0pheU43NWJwRWVSZklWdzQ4TlhUUT09&bul_id=MDMxdHZjWTk1SjFzTzNkRXYzcVZjdz09 | 2010 |
| Mali | IPUMS | 2009 | Mali NSO Link:http://www.instat-mali.org/contenu/eq/rana16pas1_eq.pdf | 2009 |
| Mexico | IPUMS | 2010, 2015 | Mexico NSO Link: https://en.www.inegi.org.mx/app/tabulados/interactivos/?px=Poblacion_01&bd=Poblacion#variables | 2010 |
| Micronesia | NHWA SoWN | 2018 | Micronesia NSO Link:https://www.fsmstatistics.fm/wp-content/uploads/2020/04/2010-Summary-Analysis-Key-Indicators.pdf (Page7) | 2010 |
| Mozambique | NHWA SoWN | 2013-2017 | Mozambique NSO Link:http://www.ine.gov.mz/iv-rgph-2017/projeccoes-da-populacao-2017-2050 | 2017 |
| Nigeria | IPUMS | 2008, 2009, 2010 | Nigeria NSO | 2012 |
| Pakistan | NHWA SoWN | 2018 | Pakistan NSO Link: http://www.pbs.gov.pk/sites/default/files/bwpsr/punjab/MUZAFFARGARH_SUMMARY.pdf | 2017 |
| Panama | IPUMS | 2010 | Panama NSO | 2010 |
| Peru | NHWA SoWN | 2013-2018 | Peru NSO | 2017 |
| Philippines | NHWA SoWN | 2017-2018 | Philippines NSO | 2015 |
| Portugal | IPUMS | 2011 | Portugal NSO | 2011 |
| Romania | IPUMS | 2011 | Romania NSO | 2011 |
| South Africa | NHWA SoWN | 2017 | South Africa NSO | 2017 |
| United Arab Emirates | NHWA SoWN | 2016-2018 | UAE NSO | 2005 |
| United States of America | IPUMS | 2005, 2010 | United States of America NSO census | 2005, 2010 |
| Uruguay | IPUMS | 2006 | Uruguay NSO | 2006 |
| Vanuatu | NHWA SoWN | 2018 | Vanuatu NSO | 2016 |
| Venezuela | NHWA SoWN | 2018 | Venezuela NSO Link:http://www.redatam.ine.gob.ve/Censo2011/index.html | 2011 |
| Vietnam | IPUMS | 2009 | Vietnam NSO | 2009 |
| Zambia | NHWA SoWN | 2017-2018 | Zambia NSO Link: https://www.zamstats.gov.zm/index.php/publications | 2018 |

* . A situational analysis of the current health workforce in the national and provincial levels - WHO EMRO June 2020


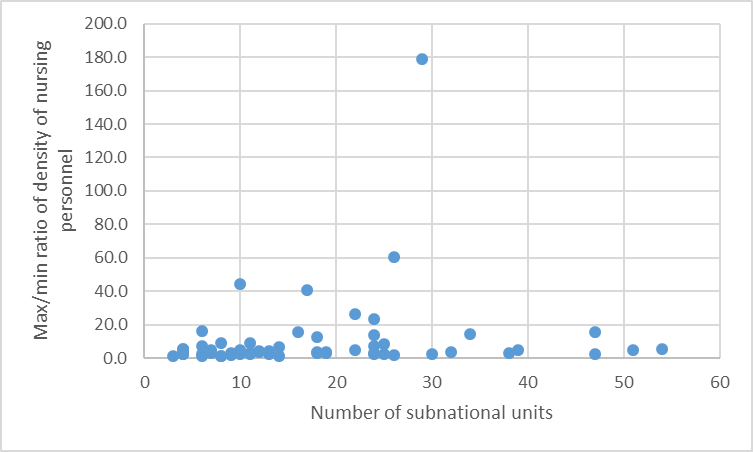


**Figure S1**. Scatter plot of amplitude of subnational variation of density (max to min ratio) and number of subnational units. Variation of density is the max to min ratio between the region with maximum density and region with lowest density.


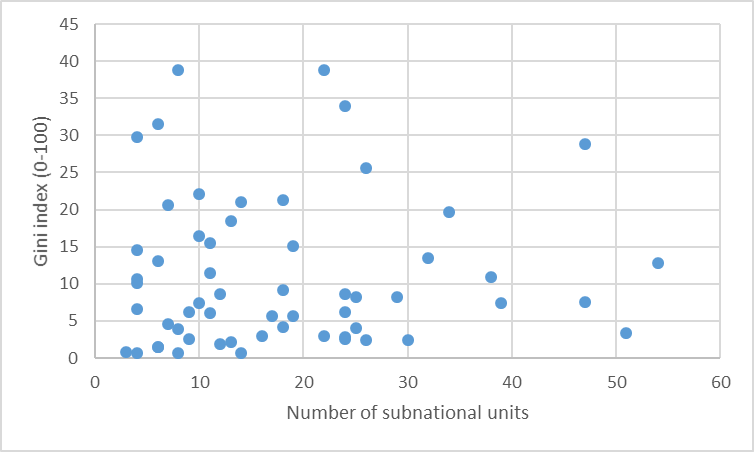


**Figure S2**. Scatter plot of Gini index measuring the inequitable distribution of nursing personnel and number of subnational units. Variation of density is the max to min ratio between the region with maximum density and region with lowest density.


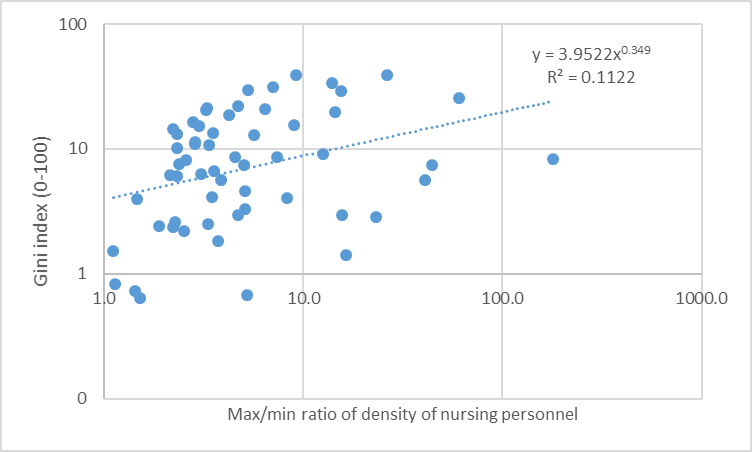


**Figure S3**. Correlation between the Gini index and the Max/min ratio of density of nursing personnel. Gini index and max/min ratio are on a log scale and the dashed line is a power function regression between the two indicators (equation and R^2^ displayed on the graph).

**Multivariate analysis**

To better understand how variation of the Gini index measure inequality in nursing distribution at subnational level relates to variation of other socio-economic variables, we fitted a multivariate beta regression model using betareg package in R. This model enables to include several factors in the same regression without risk of collinearity in the measurement of strength of association.

Results of the model and variables are included in table S2.

**Table S2. Results of the multivariate beta regression of the Gini index measuring inequality in nursing distribution at subnational level with several socio-economic factors**

| **Socio-economic factors** | **Estimate** | **Standard error** | **P-value** |
| --- | --- | --- | --- |
| *Intercept of the regression* | -3.2e+00 | 2.7e+01 | 0.90 |
| **Education factors** |  |  |  |
| Inequality in education (%) | 4.2e-02 | 6.7e-02 | 0.54 |
| Inequality adjusted education index | 7.1e+00 | 6.6e+00 | 0.28 |
| Students enrolled in tertiary education | 0.0e+00 | 0.0e+00 | 0.15 |
| Gross Enrollment Ratio Primary education (female) | -1.8e-02 | 9.2e-03 | 0.05 |
| Expenditure by level of education tertiary (%) | -2.0e-02 | 1.1e-02 | 0.08 |
| **Economic and inequality indicators** |  |  |  |
| Human Development Index (HDI) | 1.4e+01 | 4.4e+00 | **<0.01** |
| Inequality adjusted HDI | -3.3e+01 | 1.6e+01 | **0.04** |
| Coefficient of human inequality | -2.0e-01 | 1.7e-01 | 0.23 |
| Inequality in income (%) | -4.3e-02 | 5.9e-02 | 0.47 |
| Inequality adjusted income index | -4.6e+00 | 6.2e+00 | 0.46 |
| Income shares held by Poorest 40% | 5.0e-01 | 7.4e-01 | 0.50 |
| Income shares held by Richest 10% | -4.1e-01 | 3.7e-01 | 0.26 |
| Income shares held by Richest % | 3.7e-02 | 4.0e-02 | 0.36 |
| Gini index on Income | 5.3e-01 | 6.2e-01 | 0.39 |
| Urban population (%) | 1.3e-02 | 7.2e-03 | 0.08 |
| **Health indicators** |  |  |  |
| Domestic Private Health Expenditure | -7.3e-03 | 8.1e-03 | 0.37 |
| Out of Pocket Health Expenditure | 1.7e-03 | 9.4e-04 | 0.07 |
| Domestic general government health expenditure as percentage of gross domestic product | 2.2e-01 | 1.1e-01 | **0.05** |
| Current health expenditure percentage of gross domestic product | -1.8e-01 | 5.6e-02 | **<0.01** |
| Inequality in life expectancy (%) | -1.4e-01 | 6.2e-02 | **0.02** |
| Inequality adjusted life expectancy index | -1.9e+00 | 5.9e+00 | 0.75 |
| Adolescent mortality rate per 100, 000 population | -3.6e-04 | 2.5e-03 | 0.89 |
| Maternal mortality ratio per 100,000 live births | 4.0e-03 | 9.8e-04 | **<0.01** |

*Note: all variables were included as continuous variable. P-value below 5% are in bold. Because of various variable definitions, the estimates and standard deviation are displayed in scientific notation with one decimal.*

The sources for each indicator included are available in table S3

**Table S3. List of variables included in the multivariate beta regression, with year, source and data link.**

| **Variables** | **Definition** | **Year** | **Source** | **Links** |
| --- | --- | --- | --- | --- |
| Students enrolled in tertiary education | Enrolment in tertiary education at each ISCED level, expressed as a percentage of total enrolment in tertiary education | 2017 | UNESCO Institute for Statistics | http://uis.unesco.org |
| Students enrolled in primary education | Enrolment in tertiary education at each ISCED level, expressed as a percentage of total enrolment in Primary education | 2017 | UNESCO Institute for Statistics | http://uis.unesco.org |
| Gross enrollment ratio - Primary (female) | Number of students (female) enrolled in a given primary school, regardless of age, expressed as a percentage of the official school-age population corresponding to the same level of education. | 2017 | UNESCO Institute for Statistics | http://uis.unesco.org |
| Gross enrollment ratio - Tertiary (female) | Number of students (female) enrolled in tertiary, regardless of age, expressed as a percentage of the official school-age population corresponding to the same level of education. For the tertiary level, the population used is the 5-year age group starting from the official secondary school graduation age. | 2017 | UNESCO Institute for Statistics | http://uis.unesco.org |
| Expenditure by level of education: tertiary (as % of government expenditure) | Total general (local, regional and central) government expenditure on education (current, capital, and transfers), expressed as a percentage of total general government expenditure on all sectors (including health, education, social services, etc.). It includes expenditure funded by transfers from international sources to government. | 2017 | UNESCO Institute for Statistics | http://uis.unesco.org |
| Human Development Index (HDI) | A composite index measuring average achievement in three basic dimensions of human development—a long and healthy life, knowledge and a decent standard of living. See Technical note 1 at http://hdr.undp.org/sites/default/files/hdr2019_technical_notes.pdf for details on how the HDI is calculated | 2018 | UNDP | https://data.un.org/ |
| Coefficient of human inequality | Average inequality in three basic dimensions of human development | 2018 | UNDP | https://data.un.org/ |
| Inequality in life expectancy (%) | Inequality in distribution of expected length of life based on data from life tables | 2018 | UNDP | https://data.un.org/ |
| Inequality-adjusted HDI (IHDI) | Inequality-adjusted HDI (IHDI): HDI value adjusted for inequalities in the three basic dimensions of human development. See Technical note 2 at http://hdr.undp.org/sites/default/files/hdr2019_technical_notes.pdf for details on how the IHDI is calculated. | 2015-2020 | UNDP | https://data.un.org/ |
| Coefficient of human inequality | Coefficient of human inequality: Average inequality in three basic dimensions of human development. | 2018 | UNDP | https://data.un.org/ |
| Inequality-adjusted life expectancy index | HDI life expectancy index value adjusted for inequality in distribution of expected length of life based on data from life tables listed in Main data sources. | 2018 | UNDP | https://data.un.org/ |
| Inequality in education (%) | Inequality in distribution of years of schooling based on data from household surveys estimated using the Atkinson inequality index. | 2018 | UNDP | https://data.un.org/ |
| Inequality-adjusted education index | HDI education index value adjusted for inequality in distribution of years of schooling based on data from household surveys listed in Main data sources. | 2018 | UNDP | https://data.un.org/ |
| Inequality in income (%) | Inequality in income: Inequality in income distribution based on data from household surveys estimated using the Atkinson inequality index. | 2010-2017 | UNDP | https://data.un.org/ |
| Inequality-adjusted income index | Inequality-adjusted income index: HDI income index value adjusted for inequality in income distribution based on data from household surveys listed in Main data sources. | 2010-2017 | UNDP | https://data.un.org/ |
| Income shares (%) held by Poorest 40 percent | Share of pre-tax national income held by the poor 40 percent of the population | 2010-2017 | UNDP | https://wid.world/fr/donnees/ |
| Income shares (%) held by Richest 10 percent | Share of pre-tax national income held by the richest 10 percent of the population. Pre-tax national income is the sum of all pre-tax personal income flows accruing to the owners of the production factors, labour and capital, before taking into account the tax/transfer system, and after taking into account the pension system. | 2010-2017 | UNDP | https://wid.world/fr/donnees/ |
| Income shares (%) held by Richest 1% | Share of pre-tax national income held by the richest 1 percent of the population. Pre-tax national income is the sum of all pre-tax personal income flows accruing to the owners of the production factors, labour and capital, before taking into account the tax/transfer system, and after taking into account the pension system. | 2010-2017 | UNDP | https://wid.world/fr/donnees/ |
| Gini Coefficient (income) | Measure of the deviation of the distribution of income among individuals or households within a country from a perfectly equal distribution. A value of 0 represents absolute equality, a value of 100 absolute inequality. | 2010-2017 | UNDP | https://data.un.org/ |
| Urban population | Urban population refers to people living in urban areas as defined by national statistical offices. The data are collected and smoothed by United Nations Population Division. | 2018 | United Nations Population Division, New York, World Urbanization Prospects: The 2018 Revision | |
| Domestic general government health expenditure (GGHE-D) as percentage of gross domestic product (GDP) (%) | Share of general government expenditures on health from domestic sources of GDP | 2017 | WHO | https://apps.who.int/gho/data/node.main.1?lang=e |
| Births attended by skilled health personnel (%) | The proportion of births attended by skilled health personnel. Numerator: The number of births attended by skilled health personnel (doctors, nurses or midwives) trained in providing lifesaving obstetric care, including giving the necessary supervision, care and advice to women during pregnancy, childbirth and the post-partum period; to conduct deliveries on their own; and to care for newborns. Denominator: The total number of live births in the same period. | 2018 | WHO | https://apps.who.int/gho/data/node.main.1?lang=e |
| Maternal mortality ratio (per 100 000 live births) | Number of maternal deaths per 100 000 live births during a specified time period, usually one year. | 2018 | WHO | https://apps.who.int/gho/data/node.main.1?lang=e |
| Out-of-Pocket-Health-Expenditure | Share of out-of-pocket payments of total current health expenditures | 2018 | WHO | https://apps.who.int/gho/data/node.main.1?lang=e |
| Current health expenditure (CHE) as percentage of gross domestic product (GDP) (%) | Level of Current Health Expenditure expressed as a percentage of GDP | 2017 | WHO | https://apps.who.int/gho/data/node.main.1?lang=e |
